# *Rosa sterilis* Juice Alleviated Breast Cancer by Triggering the Mitochondrial Apoptosis Pathway and Suppressing the Jak2/Stat3 Pathway

**DOI:** 10.3390/nu16162784

**Published:** 2024-08-21

**Authors:** Wenxi Wang, Shaolin Huang, Sha Li, Xingjie Li, Yihan Ling, Xiaomeng Wang, Shuwen Zhang, Dingzi Zhou, Wenya Yin

**Affiliations:** West China School of Public Health and West China Fourth Hospital, Sichuan University, 17# 3rd Section, Ren Min South Road, Chengdu 610041, China; 2023224045152@stu.scu.edu.cn (W.W.); huangshaolin@stu.scu.edu.cn (S.H.); outlook_7f428506f845aeb6@outlook.com (S.L.); lixingjie6588@163.com (X.L.); lingyihan@stu.scu.edu.cn (Y.L.); wangxo0325@stu.scu.edu.cn (X.W.); 2022224045149@stu.scu.edu.cn (S.Z.)

**Keywords:** *Rosa sterilis* juice, breast cancer, mitochondrial apoptosis pathway, Jak2/Stat3

## Abstract

*Rosa sterilis* (RS) is a characteristic fruit in southwestern China that has numerous health benefits; however, its pharmacological effect needs further clarification, especially with respect to the exploration of its potential anti-breast-cancer effect, as there are still knowledge gaps in this regard. This study was designed to investigate the protective effects of *Rosa sterilis* juice (RSJ) on breast cancer (BC) through in vitro cellular experiments and by establishing mouse 4T1 breast xenograft tumors. This study also had the aim of elucidating RSJ’s underlying mechanisms. RSJ can inhibit cell proliferation, affect cell morphology, and impact the clone formation ability of BC; furthermore, it can promote apoptosis by triggering the mitochondrial apoptosis pathway. In mouse 4T1 breast xenograft tumors, RSJ markedly inhibited tumor growth, relieved the pathological lesions, lowered the expression of Ki67, and regulated the expression of the apoptosis-associated protein. Moreover, we observed that RSJ can inhibit the Jak2/Stat3 signaling pathway both in vivo and in vitro. Overall, our research reveals that RSJ can alleviate BC by triggering the mitochondrial apoptosis pathway and suppressing the Jak2/Stat3 pathway, providing new dietary intervention strategies for BC.

## 1. Introduction

Breast cancer (BC), a malignant tumor, is a cancer with a high incidence and mortality rate among women. The number of BC cases is increasing, and in 2020, it accounted for around 11.7% of all new cancer cases, overtaking lung cancer as the cancer with the highest incidence worldwide [1]. The pathogenesis of BC remains to be clarified; age, family history, reproductive factors, estrogen levels, and lifestyle are factors related to BC [2]. According to molecular heterogeneity, breast cancer is usually classified into four types: Luminal A, luminal B, human epidermal growth factor receptor type 2 (HER2) overexpression, and triple-negative breast cancer (TNBC) [3], among which TNBC is the most aggressive subtype, accounting for 15–20% of cases [4,5]. Three types of cells were selected for the in vitro study: murine-derived breast cancer cell 4T1, which is most commonly used to establish a mouse breast cancer hormonal tumor model; the human-derived MCF-7 cell line, which is frequently used in studies of ER/PR-positive breast cancer; and MDA-MB-231, the most invasive human-derived TNBC cell [6]. The treatment and prognosis of BC vary substantially with the different molecular subtypes [7]. Surgery, chemotherapy, radiation, targeted therapy, immunotherapy, and hormone therapy are the primary therapeutic options for BC [8]. Although these treatments can significantly improve patient prognosis and survival, they all have varying degrees of toxicity and differing side effects [9], so it is critical to identify treatment methods that are highly efficient, have low toxicity, and have few side effects.

Dietary natural products are widely sourced and easily accessible, with multiple targets, multiple pathways, broad pharmacology, and few adverse effects [10,11]; they exhibit tremendous potential to be used in BC treatment, as they inhibit proliferation, promote apoptosis, and affect the cell cycle [12,13]. Additionally, low BC incidence and a good prognosis of BC are linked to the sustained intake of certain fruits and vegetables [12].

*Rosa roxburghii* tratt (RRT), also known as “Cili” in Chinese folklore, is a kind of medicinal and edible plant that has been consumed throughout history, and it is mainly produced in the Yunnan, Guizhou, and Sichuan regions of China [14]. RRT-derived fruit has a high nutritional value, as it is rich in vitamins, amino acids, minerals, microelements, and other biological bioactive components, such as polysaccharides, phenols, and triterpenes [15]. These rich bioactive components mean that RRT has detoxification, anti-oxidation, anti-tumor [16], and anti-hyperlipidemia effects [17]. Previous studies have found that RRT extract can inhibit the proliferation of human BC cells [9]; however, research exploring the use of RRT in vitro is scarce, and its underlying mechanism needs to be uncovered. *Rosa sterilis* (RS) is a new variety of RRT whose components are similar to those of RRT. It has fewer thorns and seeds, less plump flesh, and a slightly sweet flavor, making it more palatable than RRT. Our preliminary study showed that RSJ is rich in vitamin C, flavonoids, polyphenols, and minerals [18], but its pharmacological activity needs to be studied further.

*Rosa sterilis* juice (RSJ) is more suited for direct consumption than RS, and it can also maximize bioactive material retention. There are no studies on the effect of RS on breast cancer prevention and treatment. Therefore, this study is intended to investigate the ameliorative effect of prickly RSJ on breast cancer and to undertake a preliminary exploration of its mechanism of action through in vivo and in vitro experiments. The present study systematically investigates the effect of RSJ on a BC control.

## 2. Materials and Methods

### 2.1. Preparation of RSJ

The RS was harvested from the Planting Base in Anshun City, Guizhou Province, China. RSJ was prepared as in [18]. The RSJ was filtered with a 0.22 μm filter membrane and stored at −80 °C for in vivo and in vitro experimental research.

### 2.2. Exploring the Protective Effects of RSJ on BC In Vitro

#### 2.2.1. Cell Culture

The human breast cancer cell lines MCF-7 and MDA-MB-231, as well as the murine mammary carcinoma cell line 4T1, were donated by Xie Yongmei’s research group at the National Laboratory of Biotherapy of Sichuan University, with culturing taking place in a constant-temperature incubator (37 °C, CO_2_ concentration of 5%). DMEM and RPMI 1640 containing 10% fetal bovine serum and 1% antibiotics (Hyclone, Logan, UT, USA) were used for cell culture.

#### 2.2.2. Cell Viability Assay

MCF-7 cells, MDA-MB-231 cells, and 4T1 cells (breast cancer cells) were seeded in a 96-well plate at densities of 3 × 10^3^–5 × 10^3^ cells per well. After the cells adhered, a culture medium that contained different concentrations of RSJ (5, 10, 20, and 40 μL/mL) was added. After culturing for 24, 48, and 72 h, we added 20 μL of MTT(3- (4,5)-dimethylthiahiazo (-z-y1)-3,5-di-phenytetrazoliumromide) solution (5 mg/mL, prepared with saline) to each well, followed by incubation for 2–4 h at 37 °C, aspiration of the culture solution, and the addition of 150 μL of DMSO. The mixture was shaken until the crystals were fully dissolved. Next, using a Microplate Reader (Multiskan GO-1510, Thermo Fisher Scientific, Cleveland, OH, USA), we measured the absorbance value at 570 nm [19].

#### 2.2.3. Cell Morphology Assay

Breast cells were seeded in a 6-well plate at densities of 1.5 × 10^5^–2 × 10^5^ cells per well. After the cells adhered, a culture medium that contained different concentrations of RSJ (5, 10, 20, and 40 μL/mL) was added. We observed the changes in cell morphology after 24 h using an optical inverted microscope (CXX41, OLYMPUS, Tokyo, Japan) [20].

#### 2.2.4. Colony Formation Assay

BC cells were seeded in a 6-well plate at densities of 600–800 cells per well. After the cells adhered, a culture medium that contained different concentrations of RSJ (5, 10, 20, and 40 μL/mL) was added, and the culture medium was changed every 3 days. When the cell colonies in the control wells reached about 80%, we discarded the supernatant and washed it twice with PBS. The cells were fixed with methanol for 10 min, and 700 μL crystal violet was added to each well for 30 min [21]. The cells were washed twice with PBS, dried, and counted.

#### 2.2.5. Apoptosis Assay Using Flow Cytometry (FCM)

The pretreatment method was similar to the cell morphology assay described in Section 2.2.3. The cells were digested with trypsin. The supernatant and digestion solution were centrifuged together. The cells were washed twice with PBS. For cell staining, we followed the Annexin V/PI double staining kit instructions (Keygen Biotech, Nanjing, China); next, we carried out flow cytometry (ACEA NovoCyte™, ACEA Biosciences, San Diego, CA, USA) to detect apoptotic cells [19].

#### 2.2.6. Determination of the Mitochondrial Membrane Potential (Δψm)

The pretreatment method was similar to the cell morphology assay described in Section 2.2.3. For cell treatment, we followed the JC-1 kit’s instructions (Solarbio, Beijing, China), and we utilized a fluorescence microscope (Nikon ECLIPSE Ti, Nikon, Tokyo, Japan) to monitor the cells’ fluorescence.

### 2.3. Exploring the Protective Effects of RSJ on BC In Vivo

#### 2.3.1. Establishment of Animal Tumor Model

This study received approval from the Ethics Committee of the West China School of Public Health, Sichuan University (grant no. Gwll2021075). Female Balb/c mice (6–8 weeks old) were provided by Weitong Lihua Co., Ltd. (Beijing, China). The animals were raised in an SPF barrier environment and quarantined for adaptive feeding for a week. Next, 1 × 10^6^ 4T1 cells were implanted into the mice to create a subcuta we carried out flow cytometry (ACEA NovoCyte™, ACEA Biosciences, San Diego, CA, USA) to detect apoptotic cells neous graft tumor model [22].

The Prevention Model included (1) a control group, (2) a model group, (3) a 2 mL/kg RSJ group (RSJ-L), (4) a 4 mL/kg RSJ group (RSJ-M), and (5) an 8 mL/kg RSJ group (RSJ-H). All mouse groups, except the control group, were injected with 4T1 cell suspension subcutaneously on the right side after 7 days of RSJ gavaging, and the control group received the same volume of PBS injection. Next, the mice were continuously treated with RSJ or saline for 14 days. The mice were sacrificed on the 22nd day. Every 3 days, the length (a, mm) and width (b, mm) of the mouse tumor and body weight were recorded. The tumor volume calculation formula was as follows: tumor volume (mm^3^) = (a^2^ × b) × 0.5.

#### 2.3.2. Hematoxylin–Eosin (H&E) Staining and Immunohistochemistry (IHC)

The tumor tissues were transparently dehydrated, fixed in paraffin, and then cut into 3 µm thick slices, which were subsequently stained with 0.5% (*w*/*v*) hematoxylin and 0.05% (*w*/*v*) eosin for H&E analysis. We used the DAB detection kit to detect the primary antibody (Ki67) [23].

#### 2.3.3. Western Blot

Radio-immunoprecipitation assay (RIPA) buffer was used to homogenize tumor tissues and cells, and the protein concentration was measured and quantified (the BCA kit, Solarbio, Beijing, China). The proteins (30–40 μg) were boiled at 100 °C for 5 min, cooled, sampled, and separated using SDS–polyacrylamide gel electrophoresis (SDS–PAGE). The membrane was then transferred via a wet transfer method using PVDF membrane, soaked in 5% skimmed milk, and blocked at room temperature for 2 h. Subsequently, the primary antibody was incubated overnight at 4 °C and washed with PBST buffer 3 times (10 min/times); the secondary antibody was incubated at room temperature for 1 h and washed with PBST buffer 3 times (10 min/times). Protein bands were detected using an electrochemiluminescence (ECL) kit (4A Biotech, Beijing, China) in a chemistry imaging system and quantified using Image J 2.x [22].

### 2.4. Statistical Analysis

Data analysis and graphing were carried out using GraphPad Prism 5 (version 5.0.1.334). Statistical significance for multiple groups was calculated using a one-way ANOVA, followed by Tukey’s test for post hoc analysis. All data are shown as mean ± SD with 95% confidence level; * *p* < 0.05, ** *p* < 0.01, and *** *p* < 0.001.

## 3. Result

### 3.1. The Anti-Breast Cancer Effect of RSJ In Vitro

#### 3.1.1. RSJ Inhibits the Proliferation of BC Cells While Promoting Their Apoptosis

**Figure 1:**
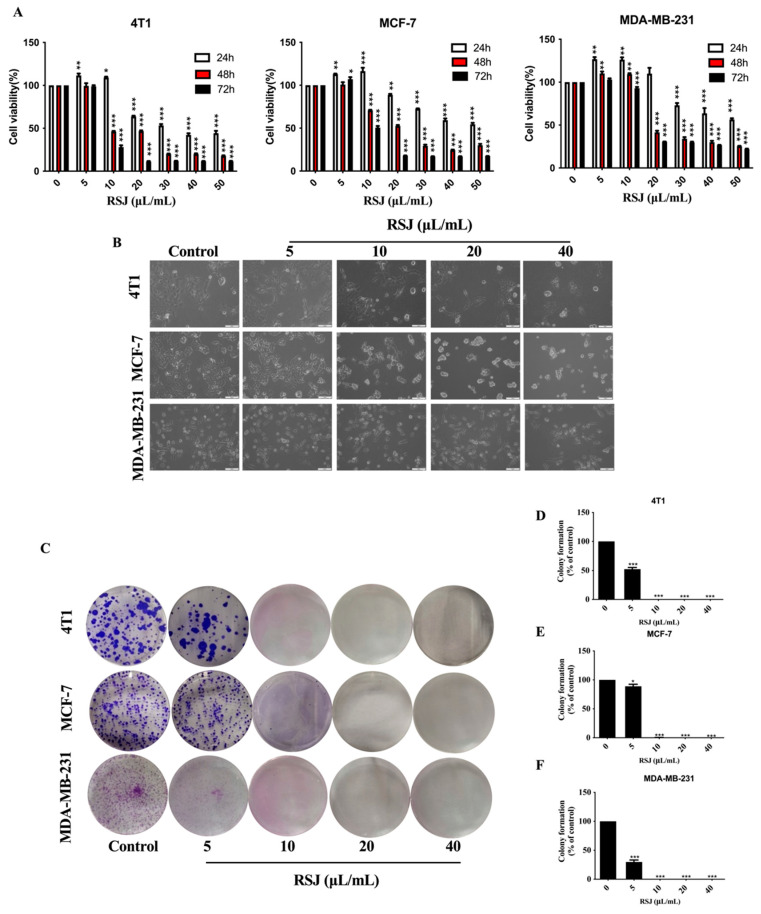
**RSJ inhibits the proliferation of breast cancer cells and alters cell morphology.** (**A**) The alterations in cell activity after RSJ treatment were measured. (**B**) The morphology of the cells, as well as the number of cells, changed after RSJ treatment (200×). (**C**–**F**) The clonogenic features of breast cancer cells following RSJ treatment.

**Figure 2:**
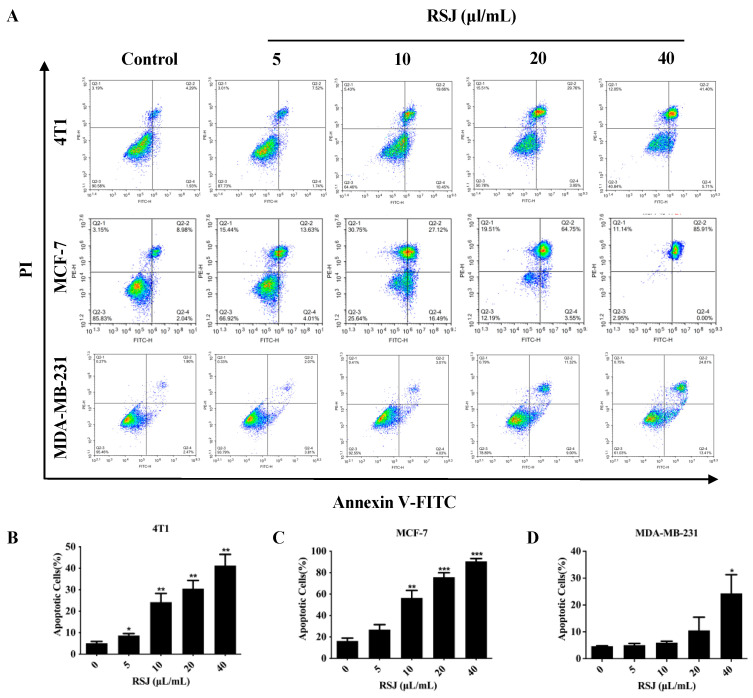
**RSJ promotes the apoptosis of breast cancer cells.** (**A**) The effect of RSJ on the apoptosis of breast cancer cells. (**B**) The apoptosis rate of 4T1 cells. (**C**) The apoptosis rate of MCF-7cells. (**D**) The apoptosis rate of MDA-MB-231 cells.

We detected the viability of the BC cells (cells derived from the murine mammary carcinoma cell line 4T1; the human breast cancer cell line MCF-7, which is commonly used in studies; and the most invasive human TNBC cell line, MDA-MB-231, were utilized) after the RSJ treatment using the MTT assay. At 5 µL/mL and 10 µL/mL, the cell viability was improved, which may have been due to the low-dose excitatory effect, but subsequently, the RSJ significantly inhibited the growth of the BC cells in a time-dependent and dose-dependent manner. After 24 h of RSJ treatment, the IC_50_ values of the 4T1, MCF-7, and MDA-MB-231 cells were 35.57 µL/mL, 49.49 µL/mL, and 50.47 µL/mL, respectively (Figure 1A); previously, we found that RSJ causes little damage to normal cells [18]. Furthermore, after the RSJ treatment, the cells clearly showed characteristics of dead cells, such as cell shrinkage, cell membrane rupture, and cell rounding. Additionally, under a microscope, we observed that the intercellular space was enlarged, that the distribution was uneven, and that there were dead cells without cell morphologies. As the concentration of RSJ increased, the number of cells gradually decreased. These phenomena were more obvious in the 4T1 and MDA-MB-231 cells (Figure 1B). A clone formation experiment was conducted to further study the inhibitory effect of RSJ on cell proliferation. The inhibition rates of the 4T1 and MCF-7 cells were both >50% after 12 days of intervention with RSJ at a concentration of 5 µL/mL. None of the three types of cells could form colonies after 12 days of treatment with RSJ at a concentration of 10 µL/mL (Figure 1C–F). The annexin V-FITC/PI double staining results (Figure 2A–D) showed that RSJ can indeed promote the apoptosis of BC cells in a dose-dependent manner. To sum up, RSJ substantially inhibits the proliferation of breast cancer cells and promotes their apoptosis.

#### 3.1.2. RSJ Triggers the Mitochondrial Apoptosis Pathway

**Figure 3:**
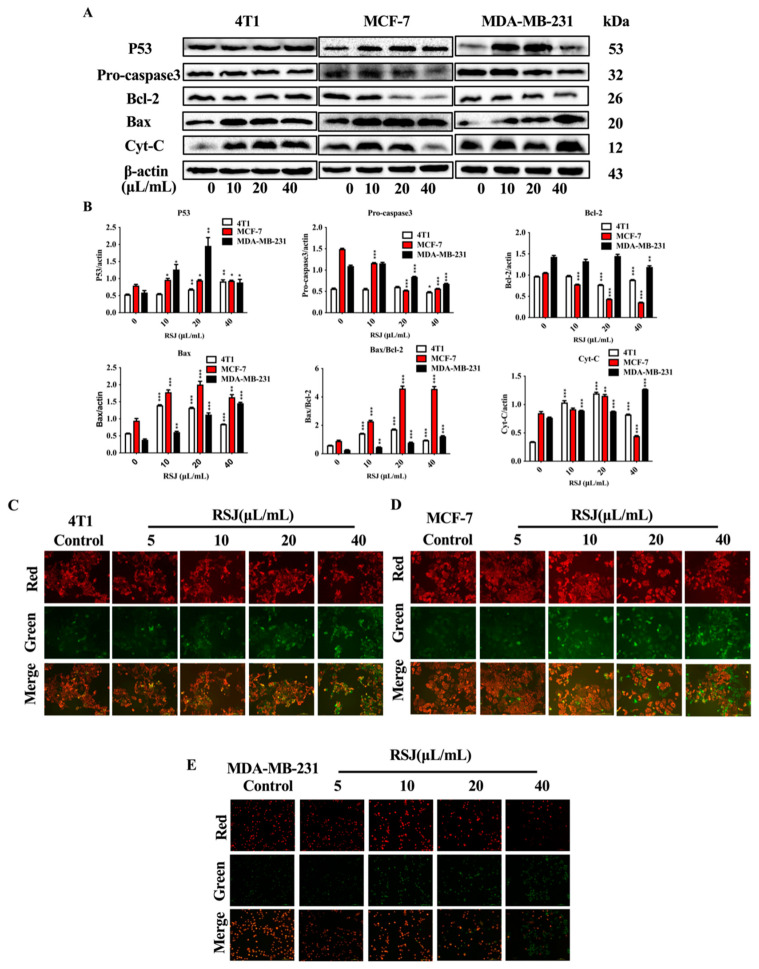
RSJ regulates the expression of the proteins in the mitochondrial pathway and reduces the mitochondrial membrane potential (ΔΨm). (**A**,**B**) Western blotting detected the expression of apoptosis-related proteins following RSJ treatment in the BC cells. (**C**–**E**) The alteration in ΔΨm in the BC cells after RSJ treatment was detected by measuring fluorescence (scale bar = 100 μm).

Mitochondria are important organelles that supply energy for cells and are involved in cell apoptosis [24]. RSJ can affect the expression levels of apoptosis-related proteins in the mitochondrial pathway after RSJ treatment. The expressions of the tumor suppressor gene P53 and pro-apoptotic protein Bax were increased significantly, and the expression of cytochrome C (Cyt-C) also increased after applying RSJ treatment to the BC cell lines (Figure 3A,B). Moreover, the expression of anti-apoptotic proteins Bcl-2 and Pro-caspase 3 decreased significantly, which led to an increase in the Bax/Bcl-2 ratio. In addition, we detected the mitochondrial membrane potential (ΔΨm). The surviving cells showed red fluorescence, while the apoptotic cells showed green fluorescence. As the concentration of RSJ increased, the green fluorescence gradually increased, while the red fluorescence gradually disappeared, showing that the ΔΨm decreased (Figure 3C–E). Therefore, we supposed that RSJ may trigger the mitochondrial apoptosis pathway to induce apoptosis.

#### 3.1.3. RSJ Suppresses the Jak2/Stat3 Signaling Pathway

**Figure 4:**
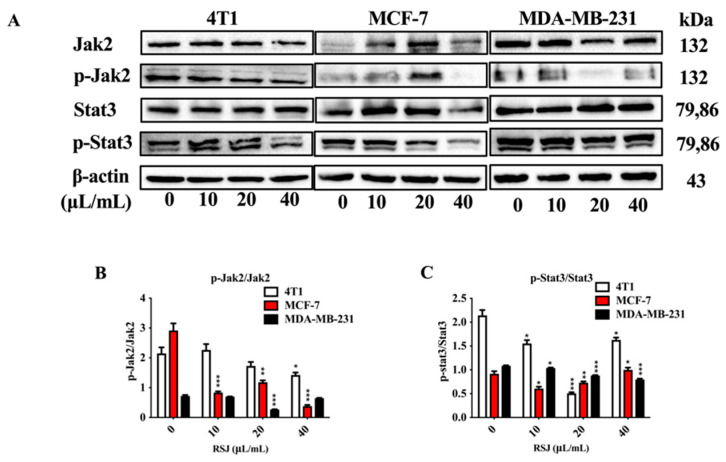
**RSJ inhibits the activation of the Jak2/Stat3 signaling pathway.** (**A**–**C**) Western blotting detected the expression of Jak2/Stat3-signaling-pathway-related proteins following RSJ treatment.

The activation of the Jak2/Stat3 signaling pathway is a key part of cell proliferation and apoptosis [25]. We detected the expression of Jak2 and Stat3 and phosphorylation in the BC cells. The RSJ decreased the p-Jak2/Jak2 and p-Stat3/Stat3 ratios (Figure 4A–C), which means that RSJ may promote apoptosis by suppressing the Jak2/Stat3 signaling pathway.

### 3.2. The Protective Effects of RSJ on BC In Vivo

#### 3.2.1. RSJ Inhibits BC Growth In Vivo

**Figure 5:**
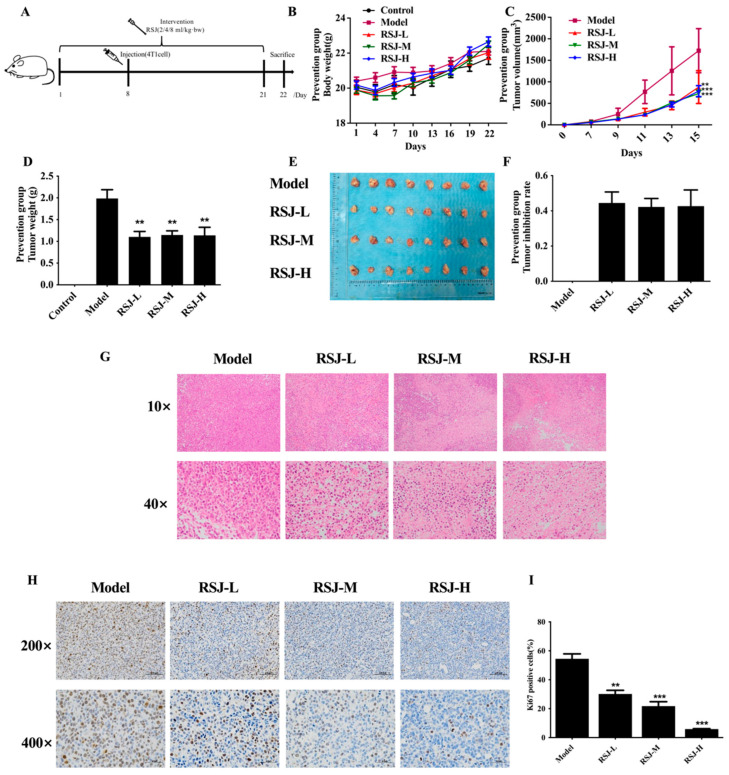
**Protective effect of RSJ on breast cancer xenograft mice.** (**A**) Experimental procedure for the xenograft mouse tumor in the Prevention Model. (**B**) Changes in body weight. (**C**,**D**) Changes in the tumor weight and volume. (**E**) Images of tumors. (**F**) Inhibition rate of mouse tumors. (**G**) Images of H&E staining of mice tumors. (**H**,**I**) Immunohistochemical results for tumor tissue.

Through our research and cell experiments, we can infer that RSJ could inhibit the proliferation of BC cells and promote their apoptosis, although the possible mechanisms underlying this need to be fully explored. In our study, we also established mouse 4T1 breast xenograft tumors to further research the protective effect of RSJ and verify its potential mechanism. Previously, we found that RSJ had no significant effect on the body weights and histological structures of various organs in healthy mice [18]. In the Prevention Model (Figure 5A), there was no significant difference in body weight between the groups during the experiment (Figure 5B). Compared with the model group, the tumor growth of the mice in all the intervention groups was slowed down, and the tumor weight and volume were significantly decreased after the treatment with RSJ (Figure 5C–E). The tumor inhibition rates (Figure 5F) in the RSJ-L, RSJ-M, and RSJ-H groups were 44.48%, 42.23%, and 42.65%, respectively. These results suggest that RSJ can inhibit tumor growth. The H&E staining showed that nuclear density was significantly lower, cytoplasmic staining vacuolation was enhanced, and tumor cell necrosis was increased in the RSJ intervention group (Figure 5G). Additionally, by carrying out IHC (Figure 5H,I), we noticed that RSJ treatment may dramatically lower the expression of Ki67, a crucial marker directly associated with proliferation in malignant tissues (*p* < 0.05). These results suggested that RSJ has a certain protective effect on BC.

#### 3.2.2. RSJ Exerts Anti-Tumor Activity by Promoting Apoptosis

**Figure 6:**
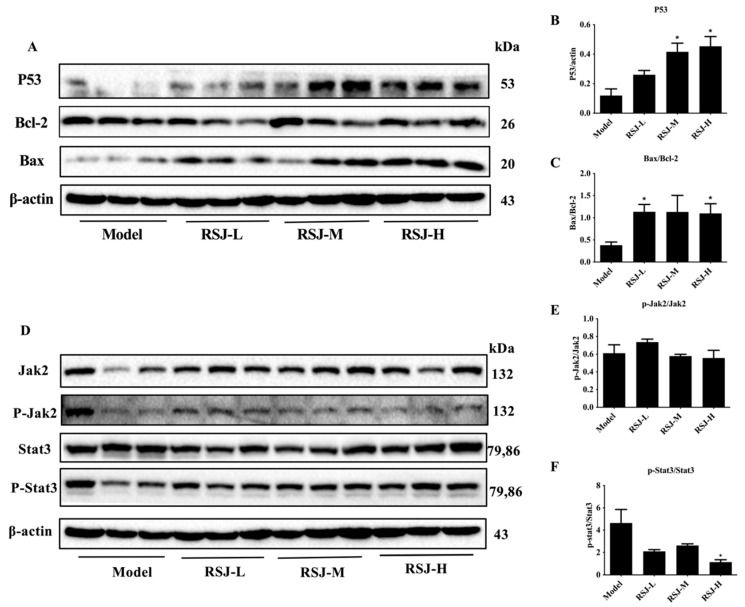
**RSJ regulates the expression of apoptosis-related proteins and suppresses the Jak2/Stat3 pathway.** (**A**–**C**) Western blotting detected the expression of the apoptosis-related proteins in tumor tissues. (**D**–**F**) Western blot detected the expression of Jak2/Stat3 signaling pathway-related proteins.

We investigated the expression levels of apoptosis-related proteins in tumor tissues. In the Prevention Model, the expression of P53 and the Bax/Bcl-2 ratio increased after the RSJ treatment compared with the model group (Figure 6A–C).

#### 3.2.3. RSJ Inhibits the Jak2/Stat3 Signaling Pathway

We also investigated the effects of RSJ on the Jak2/Stat3 signaling pathway in tumor tissue. In the Prevention Model, the p-Stat3/Stat3 ratio tended to decrease after the RSJ treatment, especially in the RSJ-H group (*p* < 0.05), while the p-Jak2/Jak2 ratio showed a decreasing trend in the RSJ-M and RSJ-H groups, but these results were not statistically different (Figure 6D–F).

## 4. Discussion

BC is one of the most common malignant tumors in the world, with a high risk of metastasis and recurrence. With the rapid development of medical technology, BC treatment has made great progress, but it inevitably has side effects, such as fatigue, nausea, vomiting, and osteoporosis-related effects [26,27]. Therefore, it is necessary to find a low-toxicity drug with few side effects. Dietary natural products, especially some phenolic-rich fruits, such as pomegranate, mangosteen, and citrus fruits, exhibit superior cytotoxicity against BC cells [12]. RS is a phenolic-rich medicinal and edible plant. In this study, we observed that RSJ has the potential to prevent BC, as it inhibited the proliferation of BC cells and the growth of 4T1 xenograft tumors.

The malignant proliferation of tumor cells is an important part of tumorigenesis and tumor development. Apoptosis is the process of programmed cell death. Inducing apoptosis is an effective method for inhibiting tumor development [28]. We found that RSJ could reduce the proliferation of BC cells and promote their apoptosis; furthermore, RSJ inhibits the growth of solid tumors and regulates the expression of apoptosis-related proteins in mouse 4T1 breast xenograft tumors.

Mitochondria are the control center of cell life activities. The mitochondrial apoptosis pathway is a classic apoptotic pathway [29]. When an apoptotic signal is received, Bax existing in the cytoplasm relocates to the surface of the mitochondria, forming a hole through the mitochondrial membrane, decreasing ΔΨm and increasing the mitochondrial membrane permeability. As a result, Cyt-C and other mitochondria-related pro-apoptotic factors are released [30], the caspase cascade is initiated, and, finally, the cells undergo irreversible apoptosis [31]. In normal circumstances, caspase3 exists in the cytoplasm in the form of Pro-caspase3 zymogen [32]. In the early stage of apoptosis, caspase3 is activated and cleaved into activated active-caspase3 [33]. A caspase cascade reaction occurs to complete signal transduction and cause an increase in active-caspase3 expression, promoting apoptosis through the mitochondrial pathway [34]. In the mitochondria-mediated apoptotic pathway, the Bcl-2 protein family plays an important role. Bcl-2 and Bax are two members of the Bcl-2 protein family that have opposite roles, as Bcl-2 plays an anti-apoptotic role, while Bax plays a pro-apoptotic role [35]. Therefore, the Bax/Bcl-2 ratio represents the direction of apoptosis [36]. The tumor suppressor gene P53 can increase the Bax/Bcl-2 ratio to promote apoptosis [37]. We observed that ΔΨm was decreased in the BC cells. Through Western blotting, we found that RSJ could increase the Bax/Bcl-2 ratio and the expression of Cyt-C and P53, reducing the expression of Pro-caspase3 in BC cells. These results suggest that RSJ induces BC cell apoptosis through the mitochondrial pathway.

**Figure 7:**
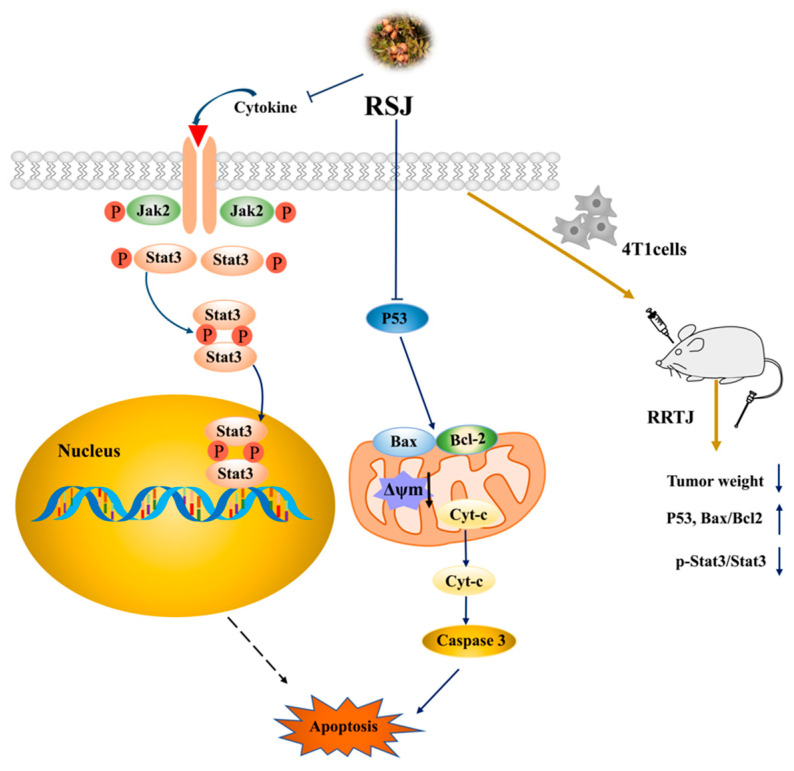
Graphical representation of RSJ’s effect on apoptosis.

The activation of the Jak2/Stat3 signaling pathway is a key factor in the occurrence of various malignant tumors [38]. After Jak2 is activated, it can promote the tyrosine phosphorylation of Stat3 into the nucleus and then specifically bind to the target promoter to promote the expression and transcription of the target genes related to the occurrence of BC, such as target genes associated with cell proliferation, survival, apoptosis, invasion, metastasis, and angiogenesis [25,39] About 70% of BC tumors show continuous Stat3 activation, and most of them are TNBC [40,41]. Activated Stat3 can upregulate the expression of anti-apoptotic factors, such as Bcl-2 and Bcl-xL, and downregulate the expression of pro-apoptotic factors, such as Bax, Bad, and Bid, to participate in tumor development [42]. In this study, we observed that the p-Jak2/Jak2 and p-Stat3/Stat3 ratios decreased; furthermore, the Bax/Bcl-2 ratio increased after the in vitro RSJ treatment. We also found the same results in tumor tissues, especially in the RSJ-H group; however, the p-Jak2/Jak2 ratio showed a decreasing trend in the RSJ-M and RSJ-H groups, although these results were not statistically different. Therefore, we can infer that RSJ may promote apoptosis and alleviate BC by suppressing the Jak2/Stat3 signaling pathway (Figure 7).

In our previous research, we conducted a thorough analysis of the composition of RSJ, which is a natural food rich in vitamin C and polyphenols, including many natural products such as caffeic acid, gallic acid, astilbin, rutin, and catechin [18]; these substances have been reported to alleviate BC. Gallic acid can treat BC by inducing G2/M phase arrest to affect the cell cycle [43]. Rutin can reduce drug resistance and the severity of chemotherapy-induced side effects in BC, and this effect may be related to its involvement in regulating the PI3K/Akt and MAPK signaling pathways [44]. Caffeic acid and gallic acid have toxic effects on MCF-7 cells and exert pro-apoptotic effects by regulating the expression of apoptosis-related genes, such as P53, Mcl-1, and P21 [45]. These abundant material bases mean that RSJ has certain anti-breast-cancer potential.

We further investigated the protective effects of RS, a new variety of RRT, on BC and preliminarily explored its mechanisms, laying the groundwork for future research on the use of RS for disease prevention and treatment. However, further investigation is needed to determine which specific bioactive compounds in RSJ contribute to its anti-breast-cancer effects; moreover, our research remains limited to the cellular and animal levels, and RSJ is yet to be evaluated in the prevention and treatment of patients with BC. Adjuvant interventions with RSJ could be considered in the future to investigate its ameliorative effects on patients. Such interventions could be of great social significance because they could reduce both the disease burden and the socioeconomic burden that BC imposes.

## 5. Conclusions

In summary, our research led to the discovery that RSJ can inhibit the proliferation of BC cells in vitro and tumor growth in vivo. Additionally, the ameliorative effects of RSJ rely on triggering the mitochondria apoptosis pathway and suppressing the Jak2/Stat3 signaling pathway. RSJ has enormous potential for application in BC treatment and is expected to become a natural dietary supplement that could be used to treat BC.

## Data Availability

The original contributions presented in the study are included in the article, further inquiries can be directed to the corresponding authors.
